# Experimental glaucoma model with controllable intraocular pressure history

**DOI:** 10.1038/s41598-019-57022-5

**Published:** 2020-01-10

**Authors:** Kayla R. Ficarrotta, Youssef H. Mohamed, Christopher L. Passaglia

**Affiliations:** 1 Chemical & Biomedical Engineering Department, University of South Florida, Tampa, FL 33620 USA; 2 Ophthalmology Department, University of South Florida, Tampa, FL 33620 USA

**Keywords:** Glaucoma, Glaucoma, Experimental models of disease, Experimental models of disease

## Abstract

Glaucoma-like neuropathies can be experimentally induced by disturbing aqueous outflow from the eye, resulting in intraocular pressure (IOP) changes that are variable in magnitude and time course and permanent in duration. This study introduces a novel method of glaucoma induction that offers researchers round-the-clock measurement and reversible control of IOP for the first time. One eye of Brown-Norway rats was implanted with a cannula tethered to a pressure sensor and aqueous reservoir. IOP was raised 10 mmHg for weeks-to-months in treated animals and unaltered in control animals. Counts of Brn3a-expressing retinal ganglion cells (RGCs) in implanted eyes were indistinguishable from non-implanted eyes in control animals and 15 ± 2%, 23 ± 4%, and 38 ± 4% lower in animals exposed to 2, 4, and 9 weeks of IOP elevation. RGC loss was greater in peripheral retina at 2 weeks and widespread at longer durations. Optic nerves also showed progressive degeneration with exposure duration, yet conventional outflow facility of implanted eyes was normal (24.1 ± 2.9 nl/min/mmHg) even after 9-weeks elevation. Hence, this infusion-based glaucoma model exhibits graded neural damage with unimpaired outflow pathways. The model further revealed a potentially-significant finding that outflow properties of rat eyes do not remodel in response to chronic ocular hypertension.

## Introduction

Glaucoma is a heterogeneous group of ocular disorders characterized by progressive and preferential loss of retinal ganglion cells (RGCs), resulting in visual field deficits and ultimately blindness. An established risk factor is high intraocular pressure (IOP) so several animal models of chronic ocular hypertension have been genetically and experimentally created to investigate causes and effects of the disease^1^. Genetic glaucoma models mainly involve select strains of mice. The most studied is the DBA/2J inbred line, which has a mutation that causes iris pigment to accumulate in the trabecular meshwork at 6–8 months of age^2^. Genetic models have the advantage that IOP increases spontaneously, allowing the application of modern molecular tools to identify critical genes and biochemical pathways of RGC death^3,4^. Disadvantages are that there is no control eye as effects are bilateral and IOP must be measured frequently as onset time is uncertain. Experimental glaucoma models span a variety of species, most popular of which are rodents and primates. Diverse methods of variable ease, reliability, and effectiveness have been employed to raise IOP by different amounts and durations. They include laser photocoagulation of trabecular meshwork^5,6^, episcleral vein cauterization^7,8^, hypertonic saline sclerosis of limbal vasculature^9^, microbead injection in the anterior chamber^10,11^, and circumlimbal sutures^12^. While more labile and laborious, experimental models have the advantage that ocular damage can be causally linked to the treatment. The non-treated eye also offers an important control for age-related loss in RGC number and function^13–15^ that can occur over long periods of IOP elevation.

A feature shared by all animal glaucoma models to date is that chronic ocular hypertension is induced by disrupting the outflow of aqueous humor through the trabecular drainage pathway of the eye. Since the ciliary body continues to form aqueous, the heightened outflow resistance causes a buildup in IOP that leads, over days to months, to patterns of retinal and optic nerve injury like that seen in glaucoma patients. The resemblance includes impaired axonal transport at the optic nerve head^16,17^, extracellular matrix remodeling of lamina cribosa^18,19^, and graded loss of large RGCs with other cell types relatively spared^20,21^. Existing glaucoma models also have important limitations by virtue of this approach. Most significant is the variable IOP history of each animal, which can range widely in magnitude and time course of elevation. Further complicating data interpretation is that IOP is usually measured by hand with a tonometer so the history of exposure is sparsely documented. Another limitation is that damage done to aqueous outflow pathways is often permanent. Relatively little is thereby known about the capacity of the eye to recover from glaucomatous insults.

Here we introduce an infusion-based model of glaucoma. The new model aims to overcome current limitations by directly pressurizing the eye through a permanently implanted cannula. By tethering the cannula to a pressure transducer and pressure source, IOP can be continuously recorded and dynamically modulated to follow any desired profile. The model was implemented in rats, and results show that chronic IOP elevation alone is sufficient to cause RGC death and optic nerve degeneration. Trabecular drainage pathways need not be perturbed genetically or experimentally. The results also show that chronic IOP elevation does not alter aqueous humor dynamics. Outflow resistance remains functionally normal in glaucomatous rat eyes, which is a first potentially-significant finding from the model.

## Results

**Figure 1:**
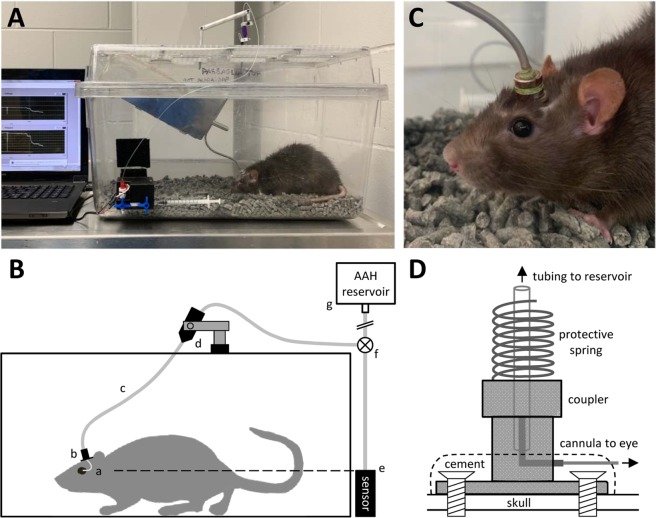
Experimental setup. (**A**) Photo of chronic eye infusion system. (**B**) Schematic of system components. Rat eye is implanted with a fine cannula (a) that is connected, via a head-mounted plastic coupler (b) and flexible tubing that runs inside a metal spring (c), to a rotary swivel (d). The swivel connects the tubing to a pressure sensor (e) and a variable-height reservoir of AAH (g) via a 3-way stopcock (f). Dashed line indicates that the sensor is positioned at rat eye level. (**C**) Photo of the head-mounted coupler. (**D**) Schematic of coupler components. Bone screws and cement affix the coupler to the skull. A metal L-shaped stent inside the coupler connects the implanted cannula to external tubing, and a metal spring attached to the coupler protects the tubing from animal bites.

**Figure 2:**
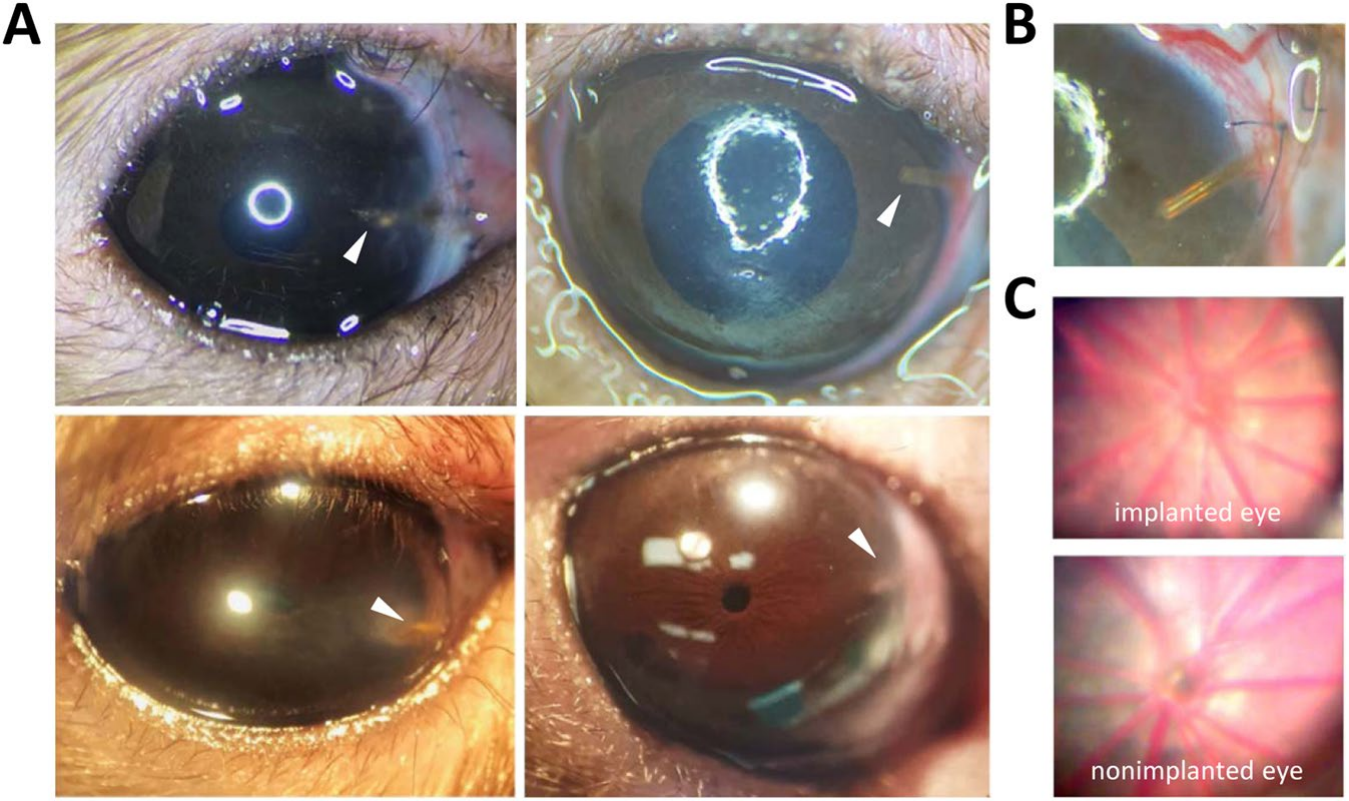
Cannula-implanted rat eyes. (**A**) Images of implanted eyes after 15 days (top left), 29 days (top right), 32 days (bottom left), and 63 days (bottom right) of IOP elevation. Arrowhead points to the cannula tip. (**B**) Close-up of cannula in the 29-day animal. (**C**) Fundus images of the implanted eye of the 63-day animal at experiment end (top) and a non-implanted eye (bottom).

**Figure 3:**
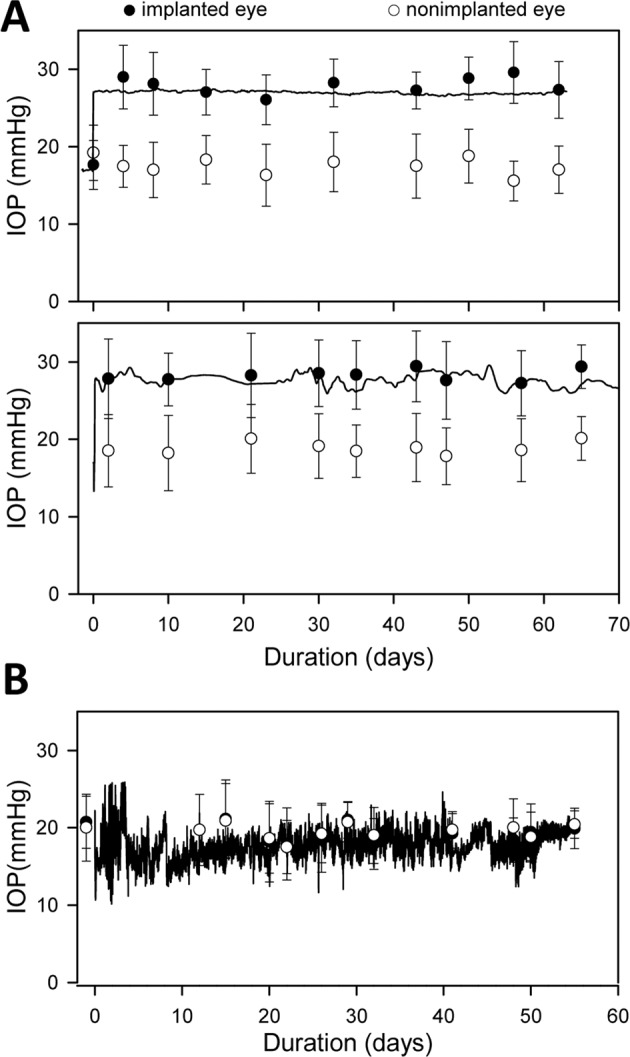
IOP history of implanted eyes. (**A**) IOP record of an eye exposed to 63 days (top) and 74 days (bottom) of infusion-induced ocular hypertension. IOP was recorded every 5 minutes by a pressure sensor connected to the cannula, which was implanted on day 0. Filled and unfilled symbols respectively plot the IOP of implanted and non-implanted eyes measured by tonometry. Error bars give standard deviation of 6 tonometer readings. (**B**) IOP record and tonometry data of a control eye that was implanted but not exposed to ocular hypertension.

A cannula was implanted in the anterior chamber of one eye of 28 rats and connected to a tethered infusion system that provides direct control of IOP. Figure 1 illustrates the experimental setup. No data are included from 4 of the animals due to severe ocular inflammation or cannula blockage during the first post-surgical week. Complications were mitigated in other animals with surgical techniques that minimized blood seepage into the anterior chamber, mechanical trauma during cannula insertion, and tissue irritation after cannula placement and with drug treatments that reduced fibrotic responses to injury (see Methods). Resting IOP was 15.6 ± 1.6 and 15.8 ± 1.5 mmHg on initial and final days of experiments, respectively, as measured by the tethered system (n = 18, p = 0.51). Figure 2 shows images of eyes implanted for weeks to months. Some fibrosis was generally observed at the cannulation site, while aqueous and vitreous fluids were otherwise clear and iridocorneal and fundus tissues looked normal. IOP was not manipulated in 6 implanted eyes, which served as experimental controls, and IOP was chronically raised 10 mmHg above the resting level in 18 implanted eyes. Figure 3 plots the IOP history of a control eye and two hypertensive eyes recorded round-the-clock with a pressure sensor connected to the cannula. Sporadic tonometer readings confirmed the prescribed IOP level was constantly applied. Since the system delivers fluid as needed to maintain the prescribed level, IOP was very stable in infused eyes. Fluctuations due to circadian rhythms^22^, ocular pulsations^23^, or other internal processes were ostensibly eliminated.

**Figure 4:**
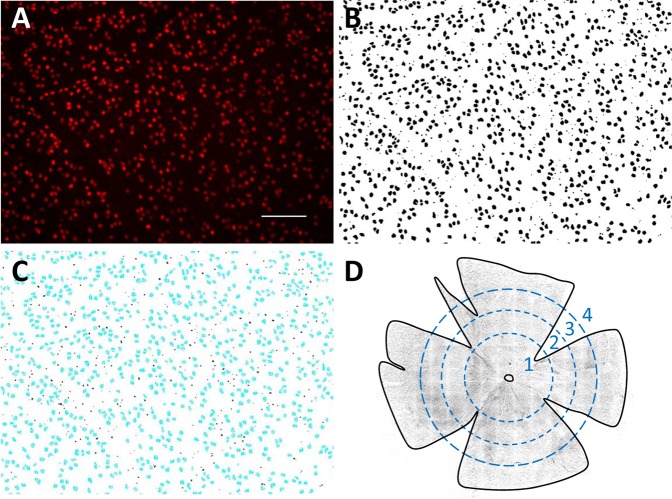
Counting retinal ganglion cells. (**A**) Raw image of Brn3a-labelled cells in a healthy rat retina. The image is a maximum-intensity projection of a stack of images collected along the z (depth) axis. Scale bar is 100 μm. (**B**) Image after applying a custom filter sequence and binary threshold to highlight labeled cells and a watershed function to separate overlapped cells. (**C**) Cells that were counted (blue) in the image using a size constraint of 15 to 300 µm^2^. (**D**) Image of a flat-mounted rat retina. Dashed circles subdivide the tissue into: (1) central, (2) inner peripheral, (3) mid peripheral, and (4) outer peripheral regions within which cell counts were tabulated. Circle radii correspond to 1.9, 2.8, and 3.7 mm in retinal space. Solid line outlines the tissue border for purpose of area measurement and cell density calculation.

**Figure 5:**
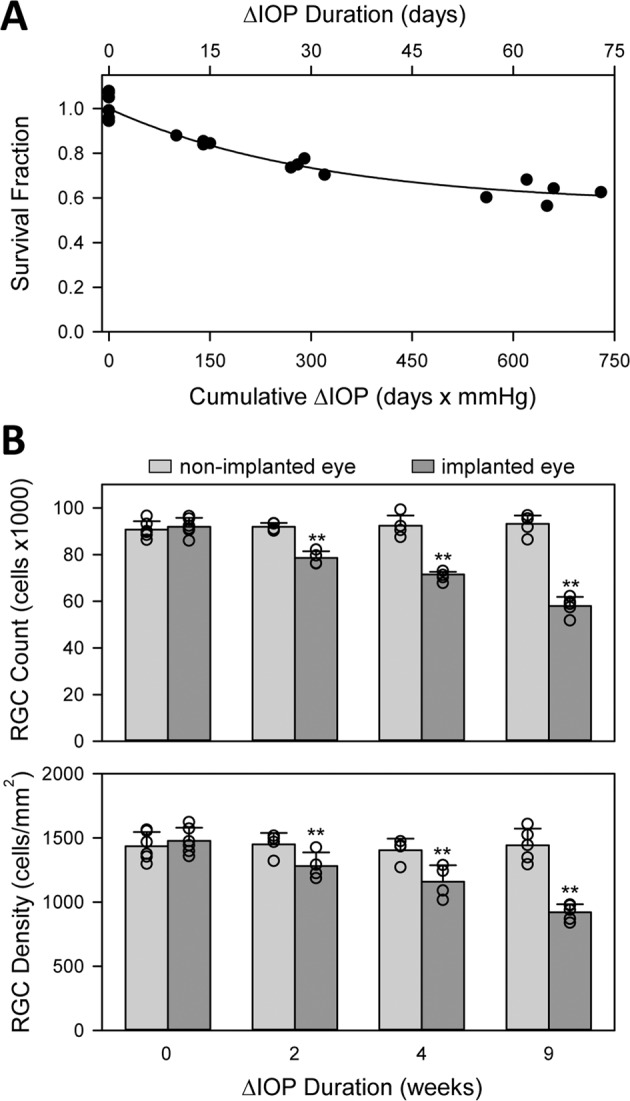
IOP-induced loss of RGCs. (**A**) Fraction of surviving cells as a function of the duration of IOP elevation and cumulative IOP insult. Survival fraction (SF) was defined as the ratio of RGC counts in the implanted and non-implanted eyes. Points at 0 days are control animals in which a cannula was implanted for several weeks but IOP was not elevated. Solid line is a regression of the data to the equation: $SF=1-b(1-{e}^{-at})$. (**B**) Average RGC count (top) and density (bottom) in non-implanted and implanted eyes exposed to 0 weeks (controls) and approximately 2, 4, and 9 weeks of IOP elevation. Symbols give individual eye data. Error bars are standard deviations. Asterisks indicate statistically significant differences.

RGCs were counted in both eyes after varying periods of IOP elevation that ranged up to 73 days. Figure 4 illustrates the cell counting procedure. RGC counts averaged 91915 ± 3740 in non-implanted eyes (n = 18 as 5 retinas were damaged during histological processing), which equated to a density of 1478 ± 102 cells/mm^2^. It was not significantly different from RGC counts in implanted eyes of control animals in which the cannula was closed off to the reservoir (92328 ± 2144, n = 4, p = 0.84) or the reservoir was set at eye level (91179 ± 7489, n = 2, p = 0.88) for 9 weeks. Hence, neither the permanent implantation of a cannula nor clamping IOP at its resting level caused measurable RGC loss. Figure 5A plots the fraction of surviving RGCs in implanted eyes against the duration of IOP elevation and against cumulative IOP insult. Survival fraction (SF) was computed from the ratio of RGC counts in the implanted and non-implanted eyes of a given animal. SF decreased with the duration of insult in a manner described by an exponential decay function: $SF=1-b(1-{e}^{-at})$. Regression of the function to the data yielded a high coefficient of determination (R^2^ = 0.94), implying that this infusion-based method of glaucoma induction produces specifiable amount of retinal damage. Based on parameter-fitted values, the rate constant of RGC loss (*a*) was 3.2% per day and the maximum inducible loss (*b*) was 44% for an IOP elevation of 10 mmHg. Figure 5B summarizes the data by clustering animals into groups that were exposed to approximately 0, 2, 4, and 9 weeks of IOP elevation. RGC counts in implanted eyes were indistinguishable from the non-implanted eye for the 0-week control group (91945 ± 3785 vs. 90641 ± 3655, n = 6, p = 0.56) and significantly less for the 2-week (78547 ± 2855 vs. 91948 ± 1617, n = 4, p < 0.01), 4-week (70557 ± 2173 vs. 92279 ± 4337 cells, n = 4, p < 0.01), and 9-week (57982 ± 3882 vs. 93142 ± 3638, n = 5, p < 0.01) groups. Analysis of RGC density yielded the same results (0 week: 1435 ± 111 vs. 1478 ± 102 cell/mm^2^, p = 0.51; 2 week: 1281 ± 106 vs. 1451 ± 89 cell/mm^2^, p = 0.04; 4 week: 1158 ± 106 vs. 1405 ± 91 cell/mm^2^, p < 0.01; 9 week: 919 ± 63 vs. 1444 ± 128 cell/mm^2^, p < 0.01).

**Figure 6:**
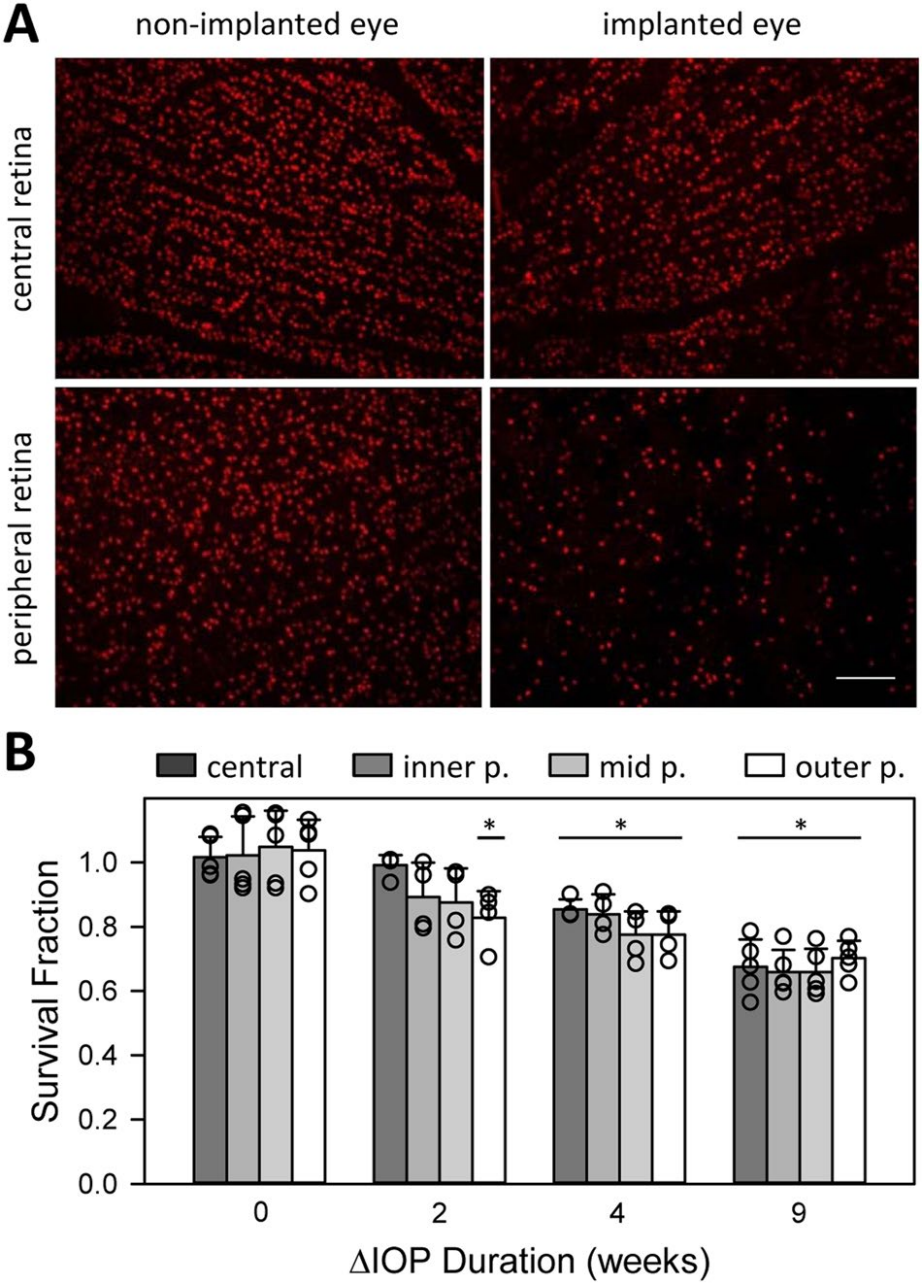
Regional variation in RGC loss. (**A**) Images of Brn3a-positive nuclei in the central (top) and peripheral (bottom) retina of the non-implanted (left) and implanted (right) eyes of an animal after 63 days of IOP elevation. Scale bar is 100 μm (**B**) Survival fraction of RGCs within central, inner peripheral, mid peripheral, and outer peripheral regions of the retina of animals exposed to 0 weeks (controls) and approximately 2, 4, and 9 weeks of IOP elevation. SF was computed for each region from the ratio of cell densities in the implanted and non-implanted eye. Symbols give individual eye data. Error bars give standard deviation. Asterisks indicate statistical differences from control animals.

RGC counts were analyzed for regional variations in cell loss. No differences were noted in SF between dorsal, ventral, nasal, and temporal retina (p = 0.93). Figure 6A indicates that RGC density was preferentially reduced in peripheral retina of several animals. The spatial gradient in cell loss was quantified by calculating RGC density for central, inner-peripheral, mid-peripheral, and outer-peripheral retina (Fig. 4D). Figure 6B plots SF for each radial region after 0, 2, 4, and 9 weeks of IOP elevation. No RGC loss was apparent in the central region of the 2-week group (p = 0.46). SF was lower on average in peripheral retina, but the reduction was significant only for the outermost region of this group (p = 0.41, 0.19, and 0.01, respectively). RGC loss was significant across the entire retina for the 4 and 9-week groups (p < 0.01).

**Figure 7:**
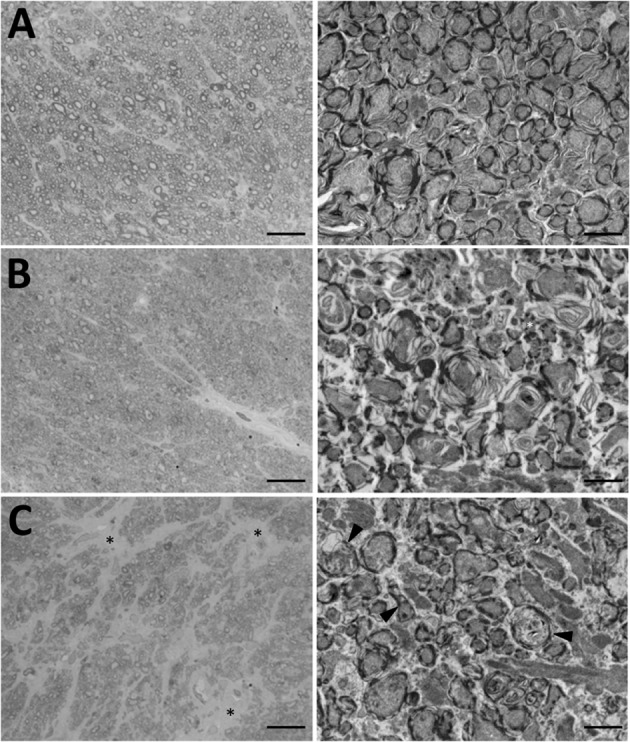
IOP-induced injury to the optic nerve. (**A**) Cross-sections of the optic nerve of the non-implanted eye of an animal. (**B,C**) Cross-sections of the optic nerve of the implanted eye of animals exposed to 2 and 9 weeks of IOP elevation, respectively. Scale bars are 100 μm in light micrographs (left) and 2 μm in transmission electron micrographs (right). Arrowheads indicate several degenerating axons with disrupted membranes and axoplasms filled with cellular debris. Asterisks mark fields of gliosis.

IOP elevation also caused optic nerve damage. Figure 7 shows micrographs of nerve cross-sections at low and high magnification from non-implanted and implanted eyes. Some reduction of axon density, disruptions of myelin wrapping, and pockets of gliosis are seen after 2 weeks of IOP elevation. The damage worsened with duration as extensive axon loss and gliosis, nerve fascicle separations, and myelin abnormalities are evident after 4 and 9 weeks of IOP elevation. Several axons can be seen filled with amorphous material from disrupted membranes as they undergo end-stage degeneration (arrowheads). The pattern of injury in these and other nerves (n = 5) resembles pressure-induced optic neuropathies of grade 3 and above observed in other rat glaucoma^24,25^.

**Figure 8:**
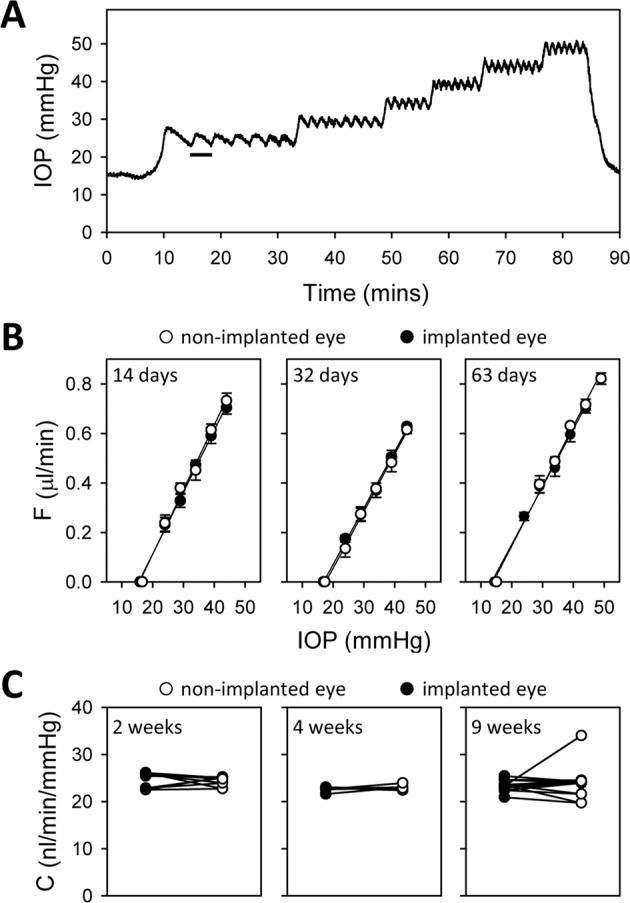
Aqueous humor dynamics of IOP-elevated eyes. (**A**) IOP record of implanted eye of an anesthetized animal during outflow facility measurement. The bar indicates one duty cycle of the pump, which turned on and off to hold IOP at different levels above the resting IOP (15 mmHg). (**B**) Fluid outflow rate (F) at different IOP set points for the implanted (filled symbols) and non-implanted (unfilled symbols) eyes of animals exposed to 2 weeks (left), 4 weeks (middle), and 9 weeks (right) of IOP elevation. F was determined from measured duty cycles in A. Lines are regression fits to estimate the data slope, which determines conventional outflow facility (**C**). C, Measured C for the implanted and non-implanted eye of all animals in each exposure group.

Aqueous humor dynamics were evaluated at experiment end with animals under terminal anesthesia. Resting IOPs of implanted and non-implanted eyes were statistically equivalent, averaging 15.4 ± 1.1 and 15.2 ± 1.1 (n = 17, p = 0.24) respectively. IOP was then set at various levels by infusing fluid with a pressure-modulated pump and eye outflow rate was measured by the pump duty cycle required to maintain each level (Fig. 8A). Figure 8B plots the outflow rate of implanted and non-implanted eyes of animals exposed to different durations of IOP elevation. Pressure-flow data were nearly identical for both eyes, indicating outflow facility was unchanged in each case. Figure 8C summarizes outflow facility estimates across animals. C averaged 24.3 ± 1.0 (n = 5), 23.0 ± 0.6 (n = 4), and 24.5 ± 4.2 (n = 8) nl/min/mmHg for 2, 4, and 9-week groups. The group averages are not different from each other (p = 0.55) nor from that of non-implanted eyes (23.5 ± 1.5 nl/min/mmHg, p = 0.76). It thus appears that sustained IOP elevation did not alter eye outflow properties.

## Discussion

This study introduces an infusion-based method of producing chronic ocular hypertension of user-defined amount and time course in rats or other animals. The method was used to raise IOP of rat eyes by 10 mmHg for up to 2 months, which caused progressive RGC death and optic nerve degeneration. The severity of damage scaled with duration in a specifiable manner, presumably due to the precise control of IOP history afforded by the permanent placement of an infusion cannula in the anterior chamber. The method does not overtly disturb aqueous outflow pathways and control experiments showed that eye cannulation does not cause retinal damage without IOP elevation, confirming that pressure alone can induce glaucoma pathologies. The method also revealed that resting IOP and conventional outflow facility were unaffected by IOP elevation even though the retina and optic nerve were severely injured.

The infusion-based glaucoma model is unique in two significant regards. Firstly, all existing animal models increase trabecular outflow resistance to reproduce open-angle forms of human glaucoma, whereas this model simulates an increase in aqueous formation rate. Hypersecretory glaucoma is extremely rare compared to the incidence of open-angle glaucoma. The condition has been associated with epidemic dropsy since dropsy patients present high IOP levels with normal outflow facility^26^. Visual field defects have been reported but they may reflect edematous effects of the disease^27^, especially since the IOP elevation is transient^28^. While an animal model of aqueous overproduction might not have compelling clinical need, it can still have translational impact by providing an alternative experimental route of understanding IOP-driven mechanisms of glaucoma. Secondly, the model offers researchers direct access and round-the-clock control of IOP for the first time. The results indicate this translates to a more reproducible glaucomatous insult across animals. It also allows for concurrent delivery of putative therapeutics into the eye, for tracking changes in outflow facility over time, and for investigating the capacity of the eye to recover from acute ocular hypertension.

The infusion-based glaucoma model has shortcomings worth noting. One is that it is limited to rats and larger animals at present. Mice eyes would require an even finer cannula, which is hard to commercially find and more susceptible to clogging. System response dynamics would also lengthen and the pressure drop across the cannula would greatly increase. A second is the surgical time and skill needed to implant and tether the cannula. It takes a few hours in rats, so microbead or suture-based models may be more tractable for large population studies. And, a third is that IOP can be measured by the system pressure sensor only when the eye is closed to the fluid reservoir. Opening the eye to a large unsealed reservoir adds considerable compliance that locks IOP at prescribed levels. The added compliance also attenuates circadian rhythms, ocular pulsations, and other internal sources of IOP fluctuation that may be important to eye mechanobiology. A recent computer simulation of the conventional outflow pathway in humans predicts that shear stress on Schlemm’s canal is highly sensitive to ocular pulsations, especially at elevated IOP^29^.

The infusion-based glaucoma model may be considered an extension of acute injury models that temporarily elevate IOP to a controlled level via needle cannulation^30–32^. The pressure insult in acute models is fairly short (hours) by comparison since the animal must be maintained under anesthesia during the cannulation procedure. Nevertheless, a single insult was found to reduce inner retina components of the electroretinogram^30–32^, alter molecular and structural responses of astrocytes and retinal neurons^31–34^, and induce focal axonal degeneration^32^ within 2 weeks of the exposure. The overall injury to the optic nerve was mild in grade, not unlike this study at the 2-week time point. One might expect the chronic model to have exhibited greater damage since IOP was elevated the entire period, but acute studies to date have applied much larger pressure levels (>50 mmHg) that may activate different neurodegenerative pathways. RGC loss observed here at 1 month (23%) is comparable to other rat glaucoma models. Studies of the microbead model report RGC losses ranging from 20 to 30% after 1 month of 10 mmHg IOP elevation (300 mmHg·days)^11,35,36^. Similar losses were reported for episcleral vein occlusion^35,36^ and hypertonic saline injection^37^ models. In some cases, greater damage was noted in peripheral retina relative to central retina as well^36^. RGC loss observed at 2 months (38%) is comparable to that reported for the vein occlusion model^35^, which also did not scale linearly with the duration of insult. Whereas, it is much less than the 50–60% loss seen in the microbead model^35,36^ and much greater than the 5–10% loss seen in a recent study that elevated intermittently elevated IOP (1 hour per day to ~35 mmHg) for 2 months with a vascular loop^38^.

An unexpected, and potentially important, result of this research was that aqueous drainage pathways continued to functional normally in glaucomatous rat eyes. It is known that trabecular tissue stiffens in human glaucoma^39–41^, owing to changes in extracellular matrix properties^42–44^ and cell mechanobiology^45^. The stiffening is thought to raise IOP by increasing outflow resistance, though experimental support is mixed to date^41,44,46^. An unresolved question is what initiates and drives the glaucomatous process. It has been hypothesized that homeostatic mechanisms maintain IOP within a healthy range by adjusting outflow resistance in response to sustained pressure changes and that glaucoma is a product of homeostatic dysfunction^47,48^. The hypothesis derives from findings that trabecular meshwork cells can sense mechanical stretch and contract^49–52^, stretch upregulates genes associated with extracellular matrix remodeling and stress signaling^49,51^, and outflow resistance depends on extracellular matrix turnover^53^ and trabecular meshwork tone^54^. A key prediction of the hypothesis is that homeostatic mechanisms should decrease outflow resistance in hypertensive eyes to reduce IOP, but the prediction has not been directly tested because existing models damage aqueous drainage pathways in order to elevate IOP and induce glaucoma and the damage may compromise homeostatic regulation. That conventional outflow facility was unaltered in this infusion-based glaucoma model suggests ocular hypertension may not be sufficient to trigger an otherwise healthy system to functionally remodel. Perhaps glaucomatous eyes develop abnormalities that heighten responsivity to IOP and stimulate an undesirable stiffening of outflow tissues.

One possibility not excluded by this study is that aqueous humor dynamics returned to normal when the tether system was disconnected at experiment end and the animal was transported to the lab for outflow facility measurement and retinal histology. Remodeling processes would have to act fairly quickly, though, if this were the case. A healthy rat eye takes around a half hour to clear a 10 mmHg increment from resting IOP, and recovery time would scale proportionately longer if outflow resistance was increased on account of the glaucomatous pressure insult applied by the tether system. It is estimated that homeostatic mechanisms would have to restore outflow tissue properties to baseline within ~1 hour to have masked any resistance change induced by chronic IOP elevation. Otherwise, pressure-flow data would have shown nonlinear behavior and outflow facility would have differed between implanted and non-implanted eyes. The possibility of rapid remodeling does not detract from the central implication of the results, which is that IOP history does not appear to have a lasting impact on aqueous humor dynamics of healthy rat eyes. Another possibility not excluded is that heightened outflow from the infusate keeps homeostatic protein concentrations from building up to levels needed to initiate remodeling.

## Methods

All experiments were conducted in accordance with the ARVO Statement for Use of Animals in Ophthalmic and Vision Research and with approval by the Institutional Animal Care and Use Committee at the University of South Florida.

### Experimental setup

Male retired-breeder Brown-Norway rats (300–400 g) were housed under a 12-hour light/12-hour dark cycle in a temperature-controlled room (21 °C) with free access to food and water. On the day of surgery, animals were anesthetized with ketamine hydrochloride (75 mg/kg) and xylazine (7.5 mg/kg) given IP as needed and rested on an isothermal pad (37 °C). The head was secured in a stereotaxic instrument, scrubbed with sterilizing agents, and opened to expose the skull. A fine polyimide cannula (ID: 0.1 mm, length: 20 mm) was passed subdermally to one eye and permanently implanted in the anterior chamber. Details of the implantation procedure have been described^22,55^ In short, the cannula was heparinized overnight and filled with an artificial aqueous humor (AAH) solution (130 mM NaCl, 5 mM KCl, 5 mM NaHCO_3_, 1 mM CaCl_2_, 0.5 mM MgCl_2_, 20 mM HEPES, pH 7.25). A translimbal hole was made in the eye with a 33 G needle, and 1 μl triamcinolone acetonide (Triesence, Alcon, Fort Worth, TX) was injected to counteract inflammatory processes. The cannula tip was then inserted through the hole into the anterior chamber, secured in place with sutures, and connected to a custom-made plastic coupler that was affixed to the skull with bone screws and cement. The coupler was tethered via a swivel mount system (Instech Laboratories, Plymouth Meeting, PA), polyethylene tubing (ID: 0.26 mm, length: 50 cm), and 3-way stopcock to a temperature-compensated pressure sensor (Model 26PC, Honeywell, Morristown, NJ) and a variable-height reservoir (250 ml) of AAH (Fig. 1). In a few experiments the coupler was tethered instead to a portable pressure-regulated AAH pump^55^. Pressure sensor output was amplified, lowpass filtered with 1-Hz cutoff frequency, and digitized at 2 Hz by a custom LabVIEW (National Instruments, Austin, TX) computer program. Sensor output was calibrated against a mercury manometer prior to study commencement and verified intermittently thereafter. The calibration procedure and hydrodynamic properties of the system have been described^56^. There is a pressure drop between the sensor and the eye due to the cannula. The steady-state difference is small (<1 mmHg) though because the cannula has a measured conductance of 1 μl/min/mmHg, which is 40-fold greater than the outflow facility of rat eyes^56^. Sensor output was recorded round-the-clock throughout the experiment in every animal, and IOP data were sporadically checked against tonometer (TonoVet, Icare USA Inc, Raleigh, NC) readings from both eyes with animals under isoflurane anesthesia. Every 12 hours for 3–5 days after cannula implantation, animals were given an intramuscular injection of carprofen (5 mg/kg) for pain relief and each eye was instilled with a drop of 1% cyclopentolate hydrochloride to prevent iris attachments and 1% prednisone acetate to combat inflammation. Implanted eyes were given 1–3 days to recover from surgery with the AAH reservoir closed. Mean daytime IOP after this period was deemed the resting level. The AAH reservoir was then opened and set at a height that raised IOP by 10 mmHg for around 0.5, 1, or 2 months. In control experiments, a cannula was implanted in the eye but IOP was not elevated. Instead, the reservoir was either closed for the experiment duration or set at eye level so that there was fluid movement but no IOP change. To assess ocular fluid clarity and vascular health, fundus pictures were acquired from some animals under acute isoflurane anesthesia by flattening the cornea of each eye with a coverslip and imaging with a stereoscope fitted with a digital camera.

### Outflow facility measurement

Aqueous humor dynamics of both eyes were evaluated in random order at the end of experiments using a modified constant-pressure perfusion technique^56^. The implanted cannula was closed and the tether system was disconnected, sealing reservoir pressure in the line while the animal was moved from housing to the laboratory. Animals were then immediately placed under ketamine/xylazine anesthesia and rested on an isothermal pad. A femoral vein catheter was inserted to steadily infuse anesthetic, and a rectal thermometer and ECG electrodes were attached to monitor body temperature and heart rate. The head was positioned in a stereotaxic instrument, and a 33 g needle was advanced into the anterior chamber of one eye. The needle was connected via polyethylene tubing to a calibrated pressure sensor and a programmable syringe pump (NE-1000; New Era Pump Systems, Farmingdale, NY) controlled by a custom program that turned flow on and off so as to maintain IOP within 2 mmHg of a user-specified set point. Once IOP settled at a resting level that fluctuated <1 mmHg over 15 minutes, the pump was instructed to raise IOP by set amounts. Net outflow rate *F* was calculated from the product of pump flow rate *F*_*p*_ (1.5 μL/min) and pump duty cycle *D*, which was measured by pump on and off duration and averaged over several cycles (i.e., $F={F}_{p}\cdot D$). Conventional outflow facility (C) was estimated by regressing *F* against IOP to quantify the slope. Data collection was then repeated on the other eye with a new needle.

### Histological processing

Animals were anesthetized with ketamine-xylazine and transcardially perfused with at least 120 ml of 10% neutral buffered formalin (NBF) until blood cleared from hepatic vasculature. Both eyes were enucleated with stumps of optic nerve and submerged in NBF for 30 minutes. Anterior tissues were excised, and posterior tissues were fixed in NBF for 48 hours. Retinas were isolated and transferred to a well plate filled with phosphate-buffered saline (PBS), and optic nerve stumps were transferred to a microcentrifuge tube filled with PBS. Retinas were processed for immunohistofluorescence by permeabilizing in 0.5% Triton X-100 in PBS (PBST) for 30 minutes at room temperature, washing in fresh 0.5% PBST, and incubating with Brn-3a (C-20) antibody (Santa Cruz Biotechnology, Dallas, TX) diluted 1:100 in blocking buffer (2% Triton X-100, 2% donkey serum, PBS) for 2 hours at room temperature and overnight at 4 °C. Retinas were washed the next day with fresh PBST and incubated in darkness with Alexa Fluor 594 Donkey anti-Goat IgG antibody (Invitrogen, Carlsbad, CA) diluted 1:500 in blocking buffer for 2 hours at room temperature. Retinas were thoroughly rinsed afterwards in PBS, cut radially at 90-degree intervals, whole-mounted flat on a charged slide with VECTASHIELD® Antifade Mounting Medium (Vector Laboratories, Burlingame, CA), and coverslipped. Prepared slides were stored in darkness at 4 °C until image acquisition. Optic nerve stumps were prepared for light microscopy (LM) and transmission electron microscopy (TEM) by removing excess sclera, washing in sodium cacodylate, immersing in 1% osmium tetroxide for 1 hour, and rinsing thrice in sodium cacodylate to remove excess osmium. Nerve stumps were dehydrated overnight in a graded series of alcohol baths, washed the next day in 100% acetone for 15 minutes, infiltrated overnight with resin diluted in a graded series of acetone baths, and heat cured in molds filled with pure resin. Embedded nerve stumps were cut into 350-nm coronal sections for LM viewing at 100x under oil immersion and into 90-nm coronal sections for TEM viewing at 10000x.

### Retinal ganglion cell counting

Brn-3a labelled retinal ganglion cells (RGCs) were counted in implanted and non-implanted eyes using a semi-automated image processing algorithm (Fig. 3A). Retina-mounted slides were viewed with a 3i spinning disk confocal microscope (Model IX81, Olympus Corporation, Tokyo, Japan). X, Y, and Z margins were identified, and a series of Z-stack images that tiled the retina was acquired with SlideBook software (Intelligent Imaging Innovations, Denver, CO). Image stacks were collapsed along a maximum-intensity Z-projection and stitched together into a single montage image of the retina. Positive-stained cells were highlighted by processing the montage image with public-domain ImageJ software (National Institute of Health, Bethesda, MD) using a custom filter sequence based on prior studies^57,58^. Binary threshold and watershed functions were then applied to separate overlapped cells, and the particle analysis function was applied to count cells (size constraint: 15–300 µm^2^). RGC counts were tabulated over the entire retina, within 4 quadrants, and for 4 annular regions representing central, inner peripheral, mid peripheral, and outer peripheral retina (Fig. 4D). Annulus boundaries were centered about the optic nerve head and set at 1.9, 2.8, and 3.7 mm in radius, as this gave similar RGC counts in each region of healthy eyes (~22000). Since the flat-mounted retina is irregular in shape, RGC counts were divided by tissue area in each region and expressed as RGC densities for analysis purposes.

### Data analysis

Statistical significance was assessed by checking for normality with a Shapiro-Wilk test and then performing one-way ANOVA or paired and unpaired t-tests at an alpha level of 0.05 using SigmaPlot software (Systat Inc, San Jose, CA). One regional cell-count dataset did not pass the normality test and significance was assessed with a Mann-Whitney rank-sum test. Results are expressed as mean ± standard deviation (SD).
